# Transferred BCR/ABL DNA from K562 Extracellular Vesicles Causes Chronic Myeloid Leukemia in Immunodeficient Mice

**DOI:** 10.1371/journal.pone.0105200

**Published:** 2014-08-18

**Authors:** Jin Cai, Gengze Wu, Xiaorong Tan, Yu Han, Caiyu Chen, Chuanwei Li, Na Wang, Xue Zou, Xinjian Chen, Faying Zhou, Duofen He, Lin Zhou, Pedro A. Jose, Chunyu Zeng

**Affiliations:** 1 Department of Cardiology, Daping Hospital, The Third Military Medical University, Chongqing, P.R. China; 2 Clinic of Health Service, Logistics Department, Air Force of Nanjing Area Command, Nanjing, Jiangsu, P.R. China; 3 Division of Nephrology, Departments of Medicine and Physiology, University of Maryland School of Medicine, Baltimore, Maryland, United States of America; University Hospital Hamburg-Eppendorf, Germany

## Abstract

Our previous study showed that besides mRNAs and microRNAs, there are DNA fragments within extracellular vesicles (EVs). The BCR/ABL hybrid gene, involved in the pathogenesis of chronic myeloid leukemia (CML), could be transferred from K562 EVs to neutrophils and decrease their phagocytic activity *in vitro*. Our present study provides evidence that BCR/ABL DNAs transferred from EVs have pathophysiological significance *in vivo*. Two months after injection of K562 EVs into the tail vein of Sprague-Dawley (SD) rats, they showed some characteristics of CML, e.g., feeble, febrile, and thin, with splenomegaly and neutrophilia but with reduced neutrophil phagocytic activity. These findings were also observed in immunodeficient NOD/SCID mice treated with K562 EVs; BCR/ABL mRNA and protein were found in their neutrophils. The administration of actinomycin D, an inhibitor of *de novo* mRNA synthesis, prevented the abnormalities caused by K562 EVs in NOD/SCID mice related to CML, including neutrophilia and bone marrow hyperplasia. As a specific inhibitor of tyrosine kinases, imatinib blocked the activity of tyrosine kinases and the expression of phospho-Crkl, induced by the *de novo* BCR/ABL protein caused by K562 EVs bearing BCR/ABL DNA. Our current study shows the pathophysiological significance of transferred tumor gene from EVs *in vivo*, which may represent an important mechanism for tumorigenesis, tumor progression, and metastasis.

## Introduction

Extracellular vesicle (EV) is an important mode of intercellular communication, besides soluble factor and tunneling nanotubule. EVs carry signals within or at their limiting membrane, providing a mechanism by which cells can exchange more complex information than previously thought [1]–[3]. Several studies have shown that in addition to proteins, mRNAs, and microRNAs, there are DNA fragments within EVs [4]–[8]. Our previous study showed the existence of DNA in EVs that could be transferred from one cell to another by endocytosis or fusion. The transferred EV DNAs have pathophysiological significance, not only to increase the DNA-coding mRNA and protein levels, but also influence the function of the recipient cells [4].

There is increasing evidence that EVs play a pivotal role in tumorigenesis, which can occur in adjacent and remote locations. EVs shed from tumor cells have the potential to increase tumor survival and expansion, independent of cell-to-cell contact. Tumor-derived EVs are fully equipped to facilitate the escape of tumor cells from immune surveillance and at the same time be involved in the establishment of an optimal environment for newly formed and metastatic tumor cells. It is interesting to find that the tumor-derived EVs could prod normal cells towards a tumor phenotype [9]. The role of the EV DNAs in this phenomenon is not clear.

As a special tumor, chronic myeloid leukemia (CML) is a clonal myeloproliferative disease, characterized by the oncogenic Philadelphia chromosome, formed by a reciprocal translocation between chromosomes 9 and 22, resulting in the novel chimeric protein BCR/ABL (breakpoint cluster region, BCR; Abelson murine leukemia viral oncogene, ABL), that dictates the pathophysiology of CML [10]–[12]. There are several pieces of evidence of increased quantity of malignant and invasive tumor-EVs in the body fluids that may contribute to the progression and metastasis in CML patients [9], [13]. Our previous *in vitro* study showed that the BCR/ABL hybrid gene could be transferred from K562 EVs to neutrophils, causing a decrease in their phagocytic activity. Whether or not the transferred BCR/ABL DNA has pathophysiological significance *in-vivo* is not known. Our present study provides the evidence that transferred EV BCR/ABL DNA has pathophysiological significance *in vivo* experiment. After injection, via tail vein, of K562 EVs into Sprague-Dawley (SD) rats or immunodeficient NOD/SCID mice for two months, the SD rats and NOD/SCID mice showed some characteristics of CML, e.g., feeble, febrile, thin, with splenomegaly and neutrophilia but with reduced neutrophil phagocytic activity. We found the BCR/ABL DNA, mRNA, and protein in the neutrophils of K562 EV-treated animals. Moreover, inhibition of *de novo* mRNA synthesis by actinomycin D prevented the features caused by K562 EVs in NOD/SCID mice, including characteristics of CML such as neutrophilia and bone marrow hyperplasia. As a specific inhibitor of tyrosine kinases, imatinib blocked the activity of tyrosine kinases and the expression of phospho-Crkl, induced by the *de novo* BCR/ABL protein caused by K562 EVs bearing BCR/ABL DNA. Our present study shows the pathophysiological significance of transferred tumor gene by EVs *in vivo*, which may represent an important mechanism for tumorigenesis, tumor progression, and metastasis.

## Materials and Methods

### 1. Cell isolation and culture

K562 and HEK293 cells were purchased from the American Type Culture Collection (ATCC; Manassas, VA, USA). These cells were cultured at 37°C in 95% air/5% CO_2_ atmosphere in Dulbecco's Modified Eagle's Medium (DMEM, Gibco, Life Technologies), supplemented with 10% fetal bovine serum (Gibco, Life Technologies).

### 2. Isolation of EVs

EVs were isolated from cell culture medium by differential centrifugation according to our previous study [4]. After removing cells and other debris by centrifugation at 500×g for 20 min, the supernatants were centrifuged again at 1,500×g for 20 min and the initial pellets were discarded. After re-centrifugation at 110,000×g for 70 min, pellets containing the EVs were resuspended in phosphate-buffered saline (PBS). The isolated EVs were then subjected to DNase I (20 Kunitz units/ml EVs) (Sigma-Aldrich Co., St. Louis, MO, USA) digestion (30 min at 37°C) to remove any DNA outside of the EVs. EDTA (10 mmol/L EVs) was then added to the EVs, and incubated for 5 min at 65°C to inactivate any residual DNase. These were the EVs used in the experiments.

### 3. DNA extraction from EVs

The isolated EVs were subjected to DNase digestion to remove the DNA exterior of the EV, as described above. Total DNA was extracted from EVs with the TIANamp Genomic DNA Kit (Tiangen Biotech Co. Ltd., Beijing, China), following the manufacturer's instructions. The DNA deposit was eluted by about 50 µl of sterile deionized water. The quality and quantity of extracted DNA were measured by spectrophotometry (A260 nm) and agarose gel electrophoresis [14].

### 4. Qualitative PCR of DNA

DNAs within EVs were amplified by qualitative PCR, using 2×Taq PCR MasterMix (Tiangen), according to the manufacturer's instructions. Twenty-five microliters of the final reaction contained 12.5 µl of 2×Taq PCR MasterMix, 1 µl of sense primer, 1 µl of antisense primer, and 10.5 µl of DNA extract. Thermocycling was conducted using a MyCycler thermal cycler (Bio-Rad Laboratories, Inc., Hercules, California, USA), initiated by a 5 min incubation at 94°C, followed by 40 cycles at 94°C for 30 sec, 58°C for 45 sec and 72°C for 45 sec. All primers used are listed in **Table 1**.

**Table 1:** Sequence of PCR amplification primers.

Primer name	Sequence (5′–3′)	Product length	Amplified gene
BCR/ABL-gD-FP	5′-TCCACTCAGCCACTGGATTTAAGCA-3′	418 bp	K562 BCR/ABL gDNA[10]
BCR/ABL-gD-RP	5′-GGTGAATTGGAAAGAAGCAGCAGGT-3′		
BCR/ABL-cD-FP	5′-CGGGAGCAGCAGAAGAAGTGT-3′	259 bp	K562 BCR/ABL mRNA
BCR/ABL-cD-RP	5′-AAAGGTTGGGGTCATTTTCAC-3′		
ACTB-cD-FP	5′- CCACGAAACTACCTTCAACTCC -3′	132 bp	Human ACTB mRNA
ACTB-cD-RP	5′- GTGATCTCCTTCTGCATCCTGT -3′		

### 5. RNA isolation and RT-PCR of mRNA

Total RNA of EVs or cells was extracted using TRIzol Reagent (Life Technologies Co., Carlsbad, California, USA). Residual DNA was removed by DNase I digestion following RNA isolation, as described below. The mixture of 11 µl purified RNA and 1 µl of 25 pmol/µl randomized primer (Toyobo Co., Osaka, Japan) in an RNase-free microcentrifuge tube was denatured by boiling at 65°C for 5 min, followed by immediate cooling on ice. Twelve µl of denatured RNA, 4 µl of 5× RT buffer (Toyobo), 2 µl dNTP mixture (10 mM each of dNTPs, Toyobo), 1 µl of 10 U/µl RNase inhibitor (Toyobo), and 1 µl ReverTre Ace (a reverse transcriptase) (Toyobo) were mixed into a total volume of 20 µl transcriptase reagents. The mixture was incubated at 30°C for 10 min, 42°C for 30 min, 85°C for 5 min, and 4°C for 5 min to allow for the synthesis of first-strand complimentary DNA (cDNA). Subsequently, qualitative PCR of cDNA was performed using 2×Taq PCR MasterMix (Tiangen) by the MyCycler thermal cycler (Bio-Rad), as described above.

### 6. Immunoblotting

The polyclonal rabbit anti-human and rat c-ABL (BCR-ABL) antibodies were purchased from Cell Signaling Technology, Inc., Danvers, MA and diluted 1∶1000 [11]. Proteins were visualized using the enhanced chemiluminescence system.

### 7. Animal experiments

SD rats or NOD/SCID mice were maintained on a 12 hr light/dark cycle in a pathogen-free animal facility at Daping Hospital. The fresh isolated EVs were injected to rats or mice every day. At 4 weeks of age, the rats were injected with PBS (200 µl), actinomycin D (7 µg/kg), K562 EVs (2×10^6^) and actinomycin D (7 µg/kg), K562 EVs (2×10^6^ EVs in 200 µl) or K562 cells (2×10^6^ cells in 200 µl) via the tail-vein. Rectal temperatures and body weights were recorded every week. After 2 months, the animals were sacrificed, the spleens were obtained, and spleen to body weight ratio was calculated. In addition, blood neutrophils were counted using Sysmex XE-2100 hematology analyzer (Sysmex Inc., Kobe, Japan). Bone marrow cells on a bone marrow smear with Wright's staining were examined under a microscope using 10×10 lens. Sections of NOD/SCID mice spleens were stained with hematoxylin-eosin (HE) and examined under a microscope using 10×40 lens.

Neutrophils were isolated from rat or mouse peripheral blood with neutrophil separating medium (TBD Co. Ltd., Tianjin, China) and then washed with PBS three times to remove any contaminating EVs. For measurement of BCR/ABL levels, genomic DNA, total RNA, and cell lysates were extracted from the neutrophils, and then analyzed by PCR, RT-PCR, and immunoblotting, as previously described [4]. All experiments were approved by the Third Military Medical University Animal Use and Care Committee.

### 8. Assay for phagocytic activity of neutrophils

Phagocytic activity of neutrophils was detected by ink phagocytosis test. After the addition of 10 µl India ink, 100 µl of heparin anti-coagulated peripheral blood were incubated at 37°C for 4 hrs and the blood smears stained with Wright's stain. To evaluate the level of phagocytic activity of neutrophils, ink-phagocytic cells and ink-particles within these cells were counted from 100 neutrophils on a blood smear with Wright's stain, under a microscope using 10×100 oil lens. Phagocytic rate (the sum of ink-phagocytic cells per 100 neutrophils) and index (the sum of ink-phagocytic particles per 100 neutrophils) were calculated.

### 9. Activity of tyrosine kinases by ELISA

Mouse neutrophils were incubated with K562 EVs (10^5^/mL) and/or imatinib (0.5 µmol/L), a specific inhibitor of tyrosine kinases, for 24 h. About 10^6^/mL of neutrophils in 100 µL PBS were collected by centrifugation at 500×g for 20 min. Then, the cell lysates of neutrophils were prepared by freeze-thawing for three times. The supernatants samples were obtained by centrifugation at 1800×g for 30 min. Tyrosine kinases activity was determined using a protein tyrosine kinases ELISA kit (R&D Systems, Inc.) according to the manufacturer's recommendation.

### 10. Expression of phospho-Crkl by immunfluorescence

The expression of phospho-Crkl in the neutrophils was detected by immunofluorescence. The polyclonal rabbit anti-mouse phospho-Crkl antibodies (Tyr207) were purchased from Cell Signaling Technology, Inc., and diluted 1∶100. Goat anti-rabbit antibodies labeled with FITC (1∶200) were binding to phospho-Crkl antibodies. Nuclei (DAPI-staining, blue) and phospho-Crkl (FITC, green) were viewed through a Zeiss LSM 510 META laser confocal microscope (Carl Zeiss) at individual excitation wavelengths (350 nm for DAPI and 490 nm for FITC). Fluorescence intensity of phospho-Crkl in neutrophils was determined under four visual fields.

### 11. Statistical analysis

The data are expressed as mean ± SD. Comparison within groups was made by ANOVA for repeated measures, and comparison among groups was made by factorial ANOVA and Duncan's test (*t*-test when only 2 groups were compared). A value of *P*<0.05 was considered significant.

## Results

### 1. Effect of K562 EVs on the pathophysiological changes in SD rats

**Figure 1:**
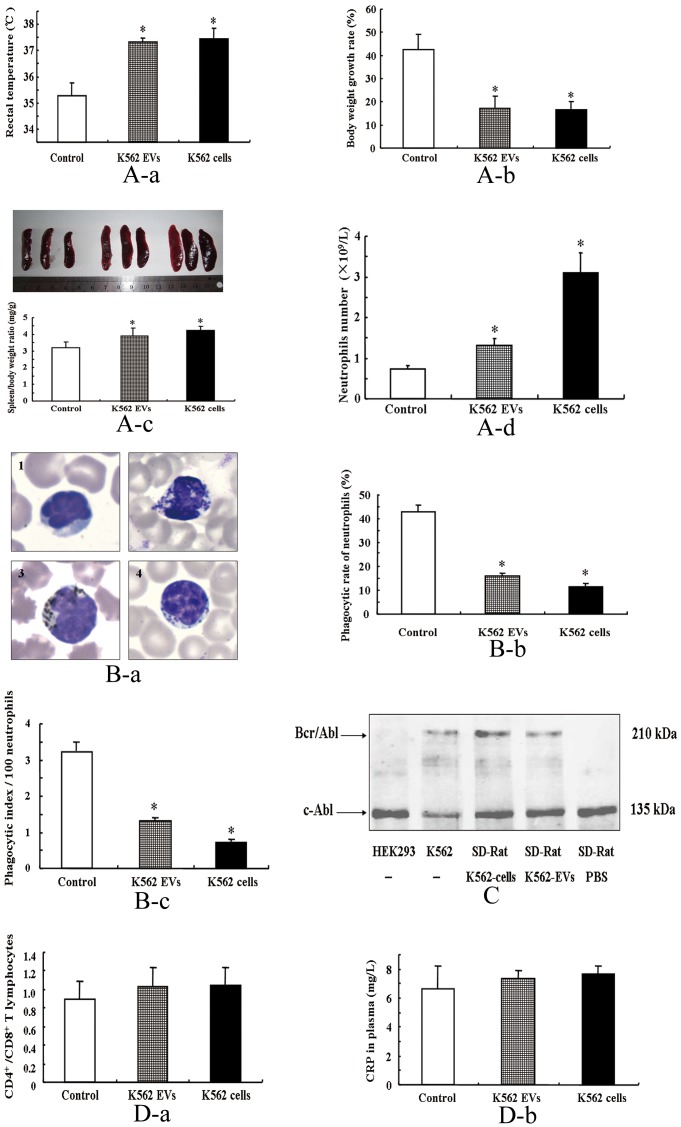
Effect of K562 EVs on several pathophysiological parameters in SD rats. (**A**): Pathophysiological parameters in SD rats bearing K562 EVs. SD rats were injected, via tail-vein, with K562 cells (2×10^6^), K562-derived-EVs (2×10^6^) or vehicle (PBS). Rectal temperature (**a**), body weight gain (**b**), spleen to body weight ratio (**c**), and neutrophils count (**d**) were obtained at 12week-old of age (* *P*<0.05 vs. control n = 3). (**B**): Phagocytic activity of neutrophils in SD rats bearing K562 EVs. (**a**): Representative images of India ink-exposed neutrophils. **1**. neutrophils from SD control rat without incubation with ink; **2**. neutrophils from SD control rat; **3**. neutrophils from K562 EVs-bearing SD rat; **4**. neutrophils from K562 cell-bearing SD rat. (**b**) and (**c**): SD rats injected via tail-vein with K562 cells (2×10^6^), K562 EVs (2×10^6^) or vehicle (PBS). Neutrophil phagocytic rate (**b**) and index (**c**) from SD rats were determined by ink phagocytosis test (* *P*<0.01 vs. control n = 3). (**C**): BCR/ABL protein expression of neutrophils in SD rats bearing K562-derived-EVs. SD rats were injected, via tail vein, with K562 cells (2x10^6^) (lane 3), K562-derived-EVs (2×10^6^) (lane 4) or vehicle (PBS) (lane 5). The neutrophils were collected and the expression of BCR/ABL protein was detected by immunoblotting. Samples from HEK293 and K562 cells were taken as negative and positive controls, respectively (lanes 1 and 2). (**D**): Immune and inflammatory responses of SD rats bearing EVs. SD rats were injected, via tail-vein, with vehicle (PBS), K562-derived-EVs (2×10^6^) or K562 cells (2×10^6^). (**a**): ratio of CD4^+^ and CD8^+^ T lymphocytes; (**b**) plasma CRP (* *P*>0.05 vs. control, n = 3).

Our previous study showed that that BCR/ABL hybrid gene could be transferred from K562 EVs to HEK293 cells or neutrophils [4]. To determine whether or not there is pathophysiological significance of the transferred BCR/ABL hybrid gene in EVs *in vivo*, we injected, via the tail vein, K562 EVs into SD rats. To decrease the immunological response due to the xenogeneic immunological incompatibility between humans and rats, we treated the SD rats with dexamethasone sodium phosphate (1.0 mg/kg/day) [15]. Two months later, SD rats, injected with K562 EVs, showed some characteristics of CML, e.g., feeble, febrile, thin, with splenomegaly and neutrophilia (**Figure 1A**), but with reduced neutrophil phagocytic activity (**Figure 1B**), similar to those seen in CML [16]. Consistent with the *in vitro* study, the neutrophils of the K562 EV-injected SD rats were found to express BCR/ABL protein (**Figure 1C**).

To determine the immune and inflammatory responses of SD rats bearing K562 cells or their EVs, we measured the ratio of CD4^+^ T lymphocytes to CD8^+^ T lymphocytes and plasma C-reactive protein (CRP) levels. We found that those above-mentioned parameters were not different among control SD rats and SD rats treated with K562 cells or K562 EVs (**Figures 1D** **-a and 1D-b**).

### 2. Effect of K562 EVs on the pathophysiological changes in NOD/SCID mice

**Figure 2:**
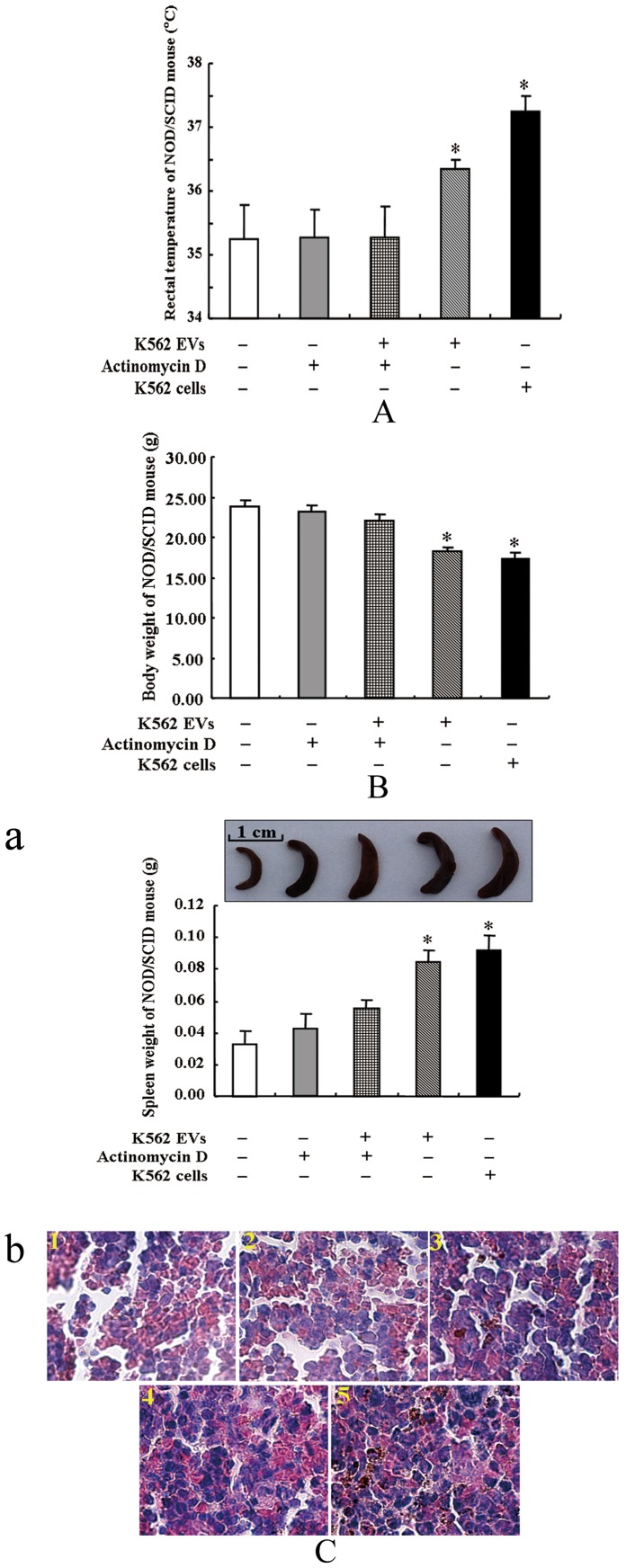
Pathophysiological parameters in NOD/SCID mice injected with K562 EVs. NOD/SCID mice were injected with K562 EVs or K562 cells and/or actinomycin D via tail-vein, every three days for 2 months. Rectal temperature (**A**), body weight (**B**), and spleen weight (**C-a**) were measured and histologies (**C-b**) were examined (* *P*<0.05 vs. control, n = 3). In **Figure C-b**, sections of spleen of NOD/SCID mouse stained with hematoxylin-eosin (HE) were examined under a microscope using 10×40 lens. Spleens of NOD/SCID mice treated with vehicle (PBS) (**1**), actinomycin D (7 µg/kg) (**2**), K562 EVs (2×10^6^) and actinomycin D (7 µg/kg) (**3**), K562 EVs (2×10^6^) (**4**), and K562 cells (2×10^6^) (**5**).

**Figure 3:**
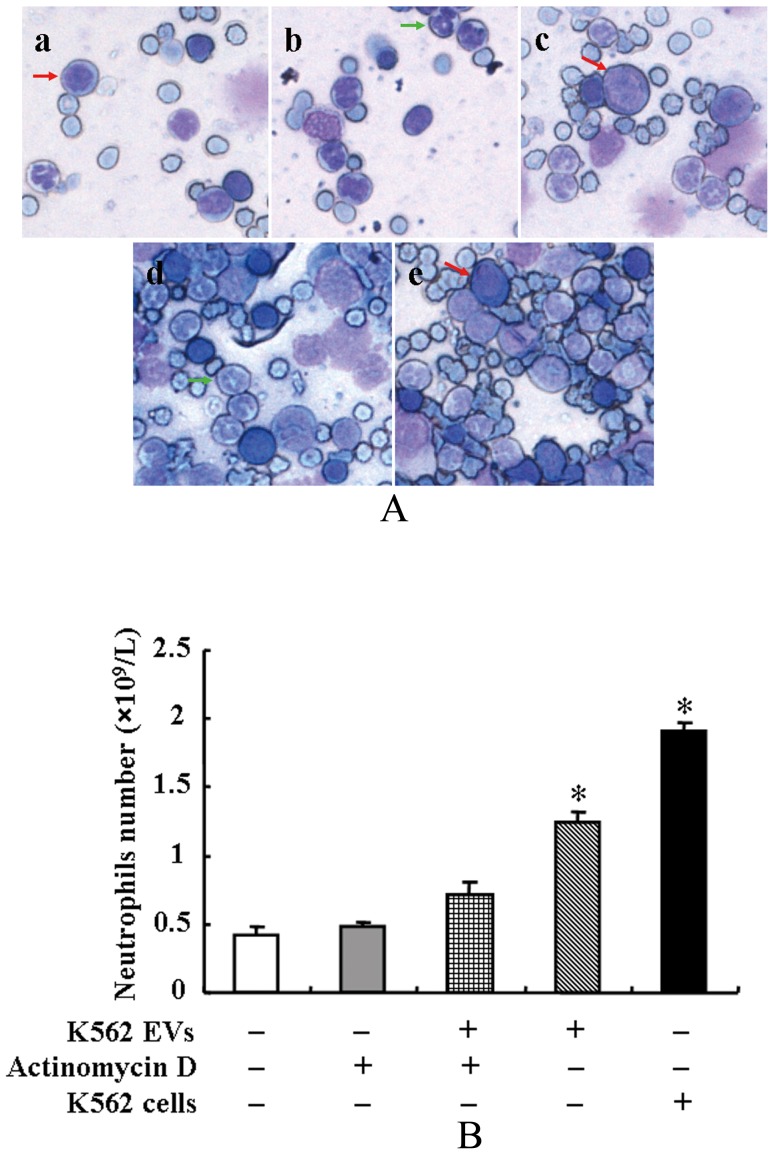
Bone marrow studies and neutrophils count in NOD/SCID mice. (**A**): Bone marrows in NOD/SCID mice. NOD/SCID mice were injected with K562 EVs or K562 cells and/or actinomycin D (7 µg/kg), via tail-vein every three days for 2 months. Wright's stained bone marrow cells were examined under a microscope using 10×10 lens. Bone marrow of NOD/SCID mice were treated with vehicle (PBS) (**a**), actinomycin D (7 µg/kg) (**b**), K562 EVs (2×10^6^) and actinomycin D (7 µg/kg) (**c**), K562 EVs (2×10^6^) (**d**), and K562 cells (2×10^6^) (**e**). Red and green arrows indicate the immature and mature granulocytes, respectively. (**B**): Neutrophils count in NOD/SCID mice. NOD/SCID mice were injected with K562 EVs (2×10^6^) or K562 (2×10^6^) cells and/or actinomycin D (7 µg/kg), via tail-vein every three days for 2 months. The number of neutrophils in the blood of the NOD/SCID mice was counted using Sysmex XE-2100 hematology analyzer (* *P*<0.05 vs. control, n = 3).

Although the SD rats were treated with dexamethasone prior to administration of the K562 EVs, it would be difficult to eliminate completely the immunological reaction due to the xenogeneic immunologically incompatible systems. Therefore, we re-performed the rat experiment in the immunodeficient mouse, NOD/SCID mouse. NOD/SCID mice were injected with K562 EVs or K562 cells and/or actinomycin D, via tail-vein, every three days for 2 months. Two months later, NOD/SCID mice, injected with K562 EVs, showed characteristics of CML similar to the SD rats injected with K562 EVs, e.g., feeble, febrile and, thin, and with splenomegaly, (**Figures 2A, 2B and 2C** **-a**). The spleens were swollen and infiltrated by leukemia cells, observed by H.E. staining (**Figure 2C** **-b**). Hyperplastic bone marrow (**Figure 3A**) and increased neutrophils count in peripheral blood (**Figure 3B**) were observed in the K562 EV-injected mice.

Our published *in vitro* study showed that the transferred BCR/ABL DNA from K562 EVs were functional, which could be transcribed into BCR/ABL mRNA and protein that subsequently affected the phagocytic activity of neutrophils. To determine whether or not the transcription of BCR/ABL gene played a key role in the pathogenesis of CML, we treated the NOD/DCID mice with actinomycin D (7.0 µg/kg), an inhibitor of *de novo* mRNA synthesis. Although actinomycin D, by itself had no effect, it blocked the development of CML caused by K562 EVs (**Figures 2A, 2B and 2C** **-a**), i.e., the hyperplastic bone marrow and neutrophilia in the K562 EVs-treated mice were no longer observed (**Figures 3A and 3B**), indicating that there was *de novo* transcription of BCR/ABL mRNA, as well as protein synthesis *in vivo*.

### 3. BCR/ABL expression in neutrophils from NOD/SCID mice

**Figure 4:**
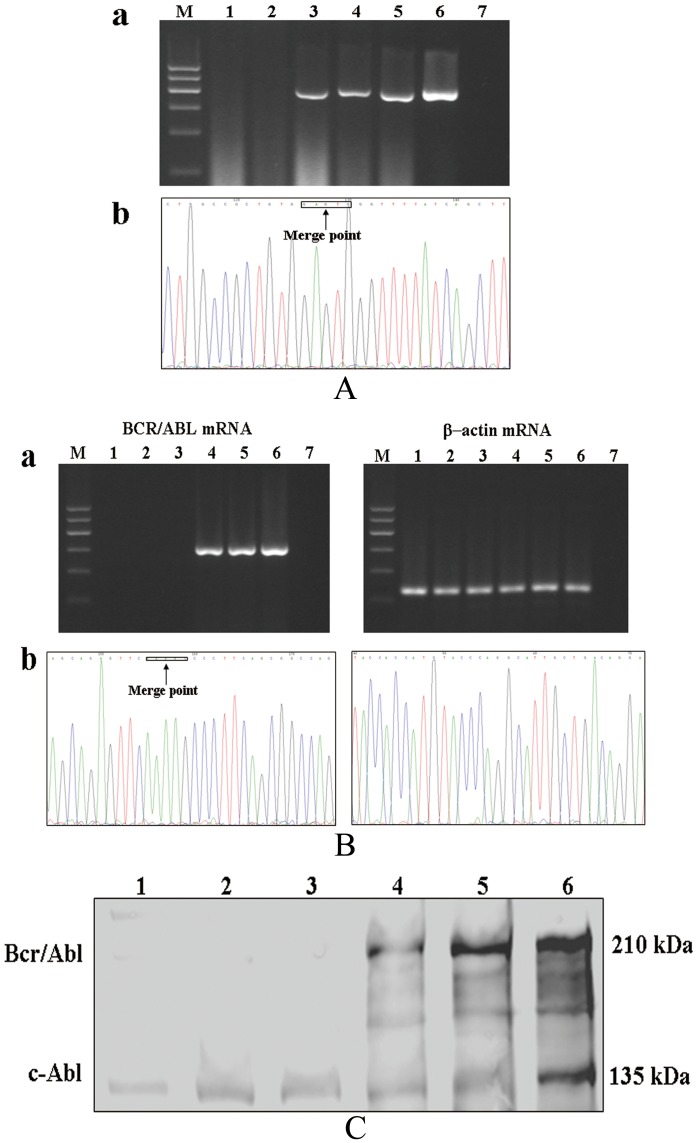
BCR/ABL expression in neutrophils from NOD/SCID mice. (**A**): BCR/ABL DNA expression in neutrophils from NOD/SCID mice. NOD/SCID mice were injected with K562 EVs or K562 cells and/or actinomycin D, via tail-vein every three days for 2 months. (**a**) The upper figure shows the BCR/ABL DNA detected by PCR that was resolved on precasted 2% agarose gel with ethidium bromide. (**b**): The lower figure shows the sequence of the PCR product of BCR/ABL DNA in the neutrophils from the NOD/SCID mice; the percentage sequence identity was more than 99.5%. The black arrow indicates the merge point of the BCR DNA and ABL DNA. (**B**): BCR/ABL mRNA expression in neutrophils from NOD/SCID mice. NOD/SCID mice were injected with K562 EVs or K562 cells and/or actinomycin D, via tail-vein every three days for 2 months. (**a**): The upper figures show the expression of BCR/ABL mRNA in neutrophils detected by RT-PCR on precasted 2% agarose gel with ethidium bromide. (**b**): The lower figures show the sequence of the RT-PCR product of BCR/ABL mRNA and β-actin mRNA in the neutrophils from the NOD/SCID mice; the percentage sequence identity is more than 99.5%. The black arrow indicates the merge point of the BCR mRNA and ABL mRNA. (**C**): BCR/ABL protein expression in neutrophils from NOD/SCID mice. NOD/SCID mice were injected with K562 EVs or K562 cells and/or actinomycin D, via tail-vein every three days for 2 months. The neutrophils were collected and the expression of BCR/ABL protein was detected by immunoblotting. **Lane M**, DNA marker; neutrophils from NOD/SCID mice treated with vehicle (PBS) (**lane 1**), actinomycin D (7 µg/kg) (**lane 2**), K562 EVs (2×10^6^) and actinomycin D (7 µg/kg) (**lane 3**), K562 EVs (2×10^6^) (**lane 4**), and K562 cells (2×10^6^) (**lane 5**); K562 cells and ddH_2_O were taken as positive and negative controls, respectively (**lane 6 and 7**).

As indicated in our published *in vitro* study, the *de novo* transcription of BCR/ABL DNA transferred by K562 EVs plays an important role in the pathogenesis of CML [4], Therefore, we examined the expressions of BCR/ABL DNA, mRNA, and protein in the peripheral blood of NOD/SCID mice injected with K562 EVs or K562 cells and found them to be expressed in their neutrophils (**Figure 4**). Actinomycin (7.0 µg/kg), by itself, had no effect on BCR/ABL DNA expression (**Figure 4A**), but it decreased the BCR/ABL mRNA and protein expressions in the neutrophils (**Figures 4B and 4C**) of K562 EV-injected NOD/SCID mice.

### 4. Inhibition of imatinib on the *de novo* BCR/ABL transcription

**Figure 5:**
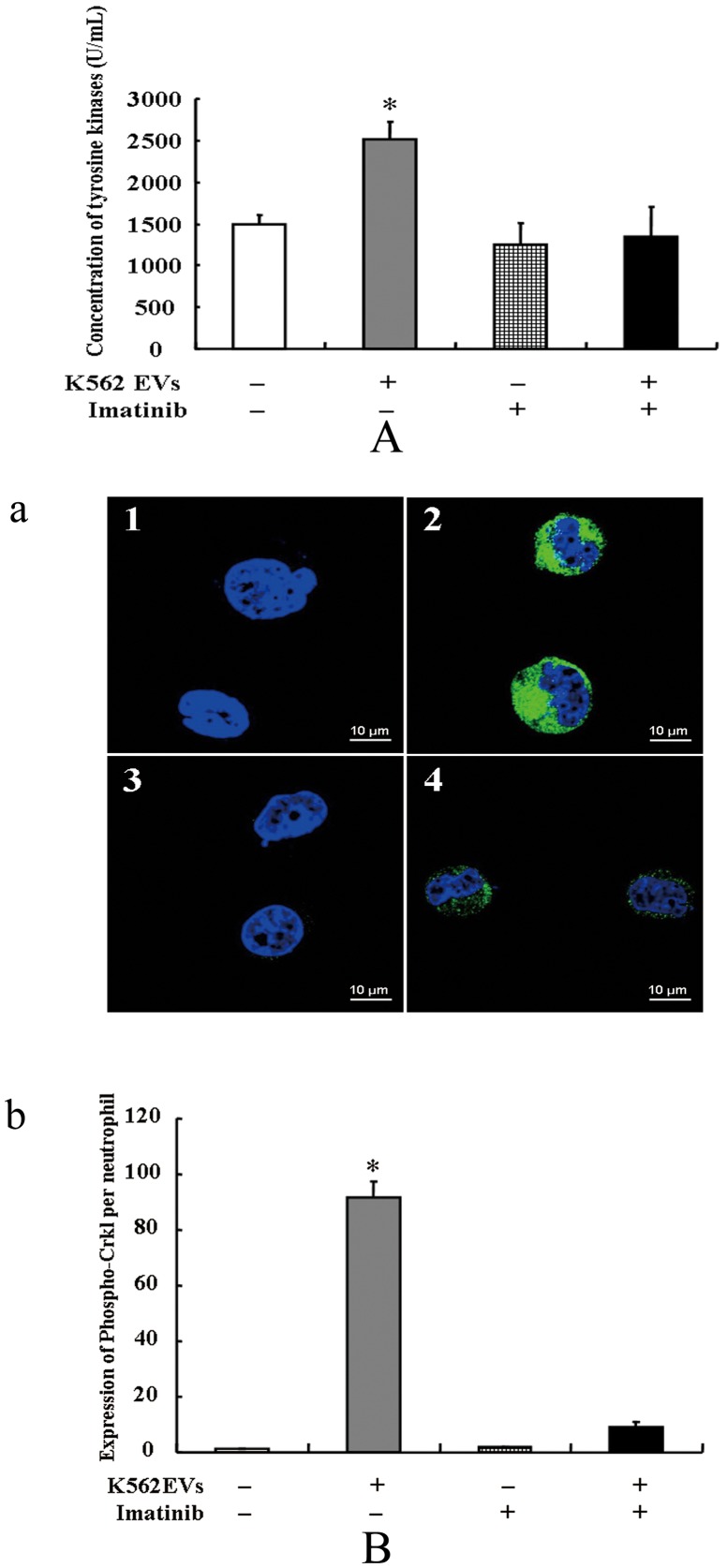
Inhibition of imatinib on the *de novo* BCR/ABL protein. (**A**): The activity of tyrosine kinases in the neutrophils. Neutrophils were incubated with K562 EVs (10^5^/mL) and/or imatinib (0.5 µmol/L) for 24 h. The activity of tyrosine kinases in neutrophils were determined by ELISA (* *P*<0.01 vs. control, n = 6). (**B**): Phospho-Crkl protein expression in the neutrophils. Neutrophils were incubated with K562 EVs (10^5^/mL) and/or imatinib (0.5 µmol/L) for 24 h. The representive images (a) and protein expression (b) of phospho-Crkl in the neutrophils were detected by immunofluorescence (* *P*<0.01 vs. other group, n = 4). Green and blue fluorescences indicate phospho-Crkl protein and Nuclei, respectively. Normal neutrophils (**1**), K562 EVs (2×10^6^) (**2**), imatinib (0.5 µmol/L) (**3**), K562 EVs (2×10^6^) and imatinib (0.5 µmol/L) (**4**).

To validate whether or not the *de novo* BCR/ABL protein influences tyrosine kinases in the neutrophils transferred by K562 EVs bearing BCR/ABL DNA, we treated these neutrophils with imatinib (0.5 µmol/L), a specific inhibitor of tyrosine kinases. We determined the activity of tyrosine kinases in neutrophils by ELISA. Although imatinib, by itself, had no significant effect, it blocked the activity of tyrosine kinases in the neutrophils transferred by K562 EVs (**Figures 5A**). As Crkl is a prominent substrate of BCR/ABL oncoprotein in CML and binds to BCR/ABL, we detected the expression of phospho-Crkl in the neutrophils by immunofluorescence. After imatinib treatment, the higher levels of phospho-Crkl in the neutrophils transferred by K562 EVs were no longer observed (**Figures 5B**), indicating the *de novo* BCR/ABL protein was functional with tyrosine kinase activity.

## Discussion

Cell to cell communication is involved in tissue morphogenesis, wound healing, and tumor metastases. Direct communication between mammalian cells occurs either by the transfer of information through EVs or physical connection through nanotubes [7], [17], [18]. Several signals via EVs can contribute to diverse cancer phenomena, such as field effect and priming of the metastatic niche [8]. For example, CML cell-derived exosomes induced angiogenic activity in endothelial cells, revealing a key role for EVs in both the pathogenesis of leukemia and its metastasis [19]. Our previous study found that the transferred BCR/ABL hybrid gene in EVs could increase the BCR/ABL mRNA and protein levels in neutrophils *in vitro* [4]. The present study showed that the BCR/ABL hybrid gene, the unique pathogenic gene causing CML, could be transferred from EVs *in vivo*, resulting in CML. These results are consistent with the view that *Plasmodium falciparum*-infected red blood cells directly communicate between parasites within a population using exosome-like vesicles that are capable of delivering genes [7].

In the present study, the initial experiment involving the injection of K562 EVs into SD rats has a limitation: a xenogeneic immunological incompatibility. Therefore, one cannot rule out that the immune response of SD rats to human antigens present in K562 leukemic cells and their EVs could have caused the CML-like symptoms, such as being feeble, febrile, thin, and with splenomegaly and neutrophilia. To overcome this limitation, we re-performed the experiment in immunodeficient NOD/SCID mice. To further confirm the transcription of EV DNAs in the recipient animal, actinomycin D, an inhibitor of *de novo* DNA transcription, was used in the *in vivo* experiment. Actinomycin D blocked the development of the characteristics of CML caused by the injection of K562 EVs in NOD/SCID mice. Therefore, the injection of K562 EVs in NOD/SCID mice caused, *in vivo*, *de-novo* BCR/ABL mRNA, and protein synthesis. However, actinomycin D is non-specific inhibitor for BCR/ABL transcription, thus we used another specific inhibitor of tyrosine kinases, which is activated by BCR/ABL protein in CML. Moreover, we also detected Crkl, which is a prominent substrate of the BCR/ABL oncoprotein in CML. As described above, imatinib blocked the activity of tyrosine kinases and the expression of phospho-Crkl, induced by the *de novo* BCR/ABL protein caused by K562 EVs bearing BCR/ABL DNA, indicated that the *de novo* BCR/ABL protein is taken in the pathogenesis of CML.

The chimeric BCR-ABL protein is involved in the pathophysiology of CML [12]. Previous study suggest that CML EVs can freely and invasively deliver BCR/ABL gene from CML cells into normal neutrophils and then influence their function in the bone marrow. Although normal EVs may instruct the bone marrow to prevent metastatic leukemia [9], the leukemic EVs can promote the mobilization of neutrophils that may play an important role in leukemic invasion and metastasis. This may explain why most hematological malignancies, especially leukemia, progress rapidly. Unfortunately, the molecular mechanisms of EV biogenesis and secretion are still not well described, but the present results suggest that genetic changes could be involved in these phenomena. It is well known that tumor EVs contribute to the horizontal propagation of oncoproteins and genetic material [8], [20]–[23]. However, our study shows that the transfer of the BCR/ABL gene from tumor-derived EVs to neutrophils may promote the invasive and metastatic process *in vivo*.

In conclusion, we have demonstrated that cell to cell communication occurs between leukemia cells and normal neutrophils and that this provides a mechanism for increasing tumorigenesis. This is a key advantage for tumor cells in maintaining invasion for survival. This process is potentially an excellent target for therapeutic approaches to block tumor progression and prevent the spread of tumor drug resistance.
